# Biological characterization of two xenografts derived from human CUPs (carcinomas of unknown primary)

**DOI:** 10.1186/1471-2407-7-225

**Published:** 2007-12-18

**Authors:** Delphine Lequin, Karim Fizazi, Saloua Toujani, Sylvie Souquère, Marie-Christine Mathieu, Pierre Hainaut, Alain Bernheim, Françoise Praz, Pierre Busson

**Affiliations:** 1 Université Paris-Sud, CNRS-UMR 8126 and Institut Gustave Roussy, 39 rue Camille Desmoulins, F-94805 Villejuif, France; 2 Department of Medicine, Institut Gustave Roussy, 39 rue Camille Desmoulins, F-94805 Villejuif, France; 3 Genomics of Cancer, FRE 2939 CNRS and Department of Medical Biology and Pathology, Institut Gustave Roussy, 39 rue Camille Desmoulins, F-94805 Villejuif, France; 4 CNRS-UPR 1983 – Institut André Lwoff, Villejuif, France; 5 Department of Medical Biology and Pathology, Institut Gustave Roussy, 39 rue Camille Desmoulins, F-94805 Villejuif, France; 6 Molecular Carcinogenesis and Biomarkers, IARC, 150 cours Albert Thomas, F-69372 Lyon, France; 7 INSERM, UMRS 762, "Microsatellite Instability and Cancer", F-75010, Paris, France; 8 Université Pierre et Marie Curie-Paris6, Centre de Recherche Saint-Antoine, Paris, France

## Abstract

**Background:**

Carcinomas of unknown primary site (CUP) are epithelial malignancies revealed by metastatic lesions in the absence of any detectable primary tumor. Although they often adopt an aggressive clinical pattern, their basic biology remains poorly understood. Laboratory research on their biology have been hampered so far by the absence of cell lines representative of CUPs.

**Methods:**

We attempted xenografts of CUP clinical specimens in immunodeficient mice and subsequent *in vitro *culture of transplanted malignant cells. Whenever possible, malignant xenografted or cultured cells were characterized by microsatellite genotyping, immunohistology, electron microscopy, multifish chromosome analysis and search of *TP 53 *gene mutations.

**Results:**

Successful xenografts were achieved in 2 cases out of 4. One of them (Capi1) was lost after 3 passages whereas the other one (Capi3) has been adapted to *in vitro *culture and is currently available to the scientific community with reliable identification based on microsatellite genotyping. Both Capi1 and Capi3 have histological characteristics of adenocarcinomas and display intense expression of EMA, CEA and cytokeratin 7. Multifish chromosome analysis demonstrated a translocation involving chromosomes 4 and 21 in both specimens. Distinct rare missense mutations of the *TP53 *gene were detected in Capi1 (codon 312) and Capi3 (codon 181); the codon 181 mutation is consistent with a previously reported similar finding in a small series of CUP specimens. Finally, intense membrane expression of c-kit was recorded in Capi3.

**Conclusion:**

Our data suggest that xenografted tumors can be obtained from a substantial fraction of CUP clinical specimens. The hypothesis of a preferential association of CUPs with *TP 53 *mutations of codon 181 deserves further investigations. The Capi3 cell line will be a useful tool for assessment of novel c-kit inhibitors.

## Background

The pathogenesis of the carcinomas of unknown primary site (CUPs) remains one of the most enigmatic topics in the field of metastasis research [1,2]. They are defined as biopsy proven metastases of a malignancy in the absence of an identifiable primary site after clinical examination, radiological imaging and biological workup. Although there is no consensus about their incidence, it is reasonable to estimate that CUPs account for 2–3 % of all newly diagnosed patients with cancer [2,3]. In all described series, this disease appears to be extremely aggressive with a median survival below 9 months [2]. Biological mechanisms underlying the CUP phenomenon remain almost entirely unknown. With regard to histological characteristics, they are predominantly classified as adenocarcinomas (50–70%) or poorly differentiated carcinomas (20–30%). Only 5–8% are squamous cell carcinomas [3]. Though CUPs comprise a heterogeneous group of tumours with widely varying natural histories, the clinical picture of CUP demonstrates common characteristics. Patients predominantly present with a short history of non-specific complaints (anorexia, weight loss, etc...). The primary tumor remains unidentified in most cases throughout the patient's life [4]. The pattern of metastatic spread tends to be different in CUPs compared to metastasized known primary tumors. Approximately 30% of patients with CUP present with three or more organs involved in contrast with less than 15% in classical metastatic syndromes [4-6]. With the exception of some treatable subgroups – for example young men with extra-gonadal germ cell tumors – patients with CUP have a very poor prognosis [7,8]. In some recent, mostly phase II studies with patients selected from poor prognostic groups, a median survival of 8–13 months was reached [9,10].

Despite these intriguing characteristics and the severity of the prognosis, biology of CUPs has been poorly investigated. Surprisingly, there are only few *in vitro *cell lines representative of this category of tumors. To our knowledge, only one such cell line is available in the American Type Culture Collection but it is uncharacterized (CRL-7431). It is worth noting that none of the NCI-60 cell line panel used for systematic *in vitro *screening of anti-cancer compounds at the National Cancer Institute (Developmental Therapeutics Program) is derived from a CUP [11]. In order to obtain biological material required for biological and pharmacological investigations of CUPs, we attempted to create xenografted tumor lines derived from fresh clinical specimens (biopsies or surgical specimens). Successful xenografts were achieved in 2 cases out of 4. One of them (Capi1) was lost after only 2 passages whereas the other one (Capi3) has been adapted to *in vitro *culture and is available to the scientific community. We here report biological characteristics of Capi3 along with some data on Capi1. Rare mutations of the *TP53 *gene were recorded for both Capi1 (exon 5) and Capi3 (exon 9).

## Methods

### Tumor transplantation in immunodeficient mice

Fragments of tumor biopsies or surgical specimens were obtained with signed informed consent from 4 patients and grafted on irradiated Swiss nude (5 Gy) and/or NOD-SCID mice. Two to six tumor fragments of about 4 mm^3 ^were implanted subcutaneously in the recipient animals.

### *In vitro *culture

A xenografted Capi3 tumor was collected and minced in small pieces in order to favor the release of tumor cells in monocellular suspension or under the form of small clumps. This cell suspension was seeded in RPMI supplemented with 20% fetal calf serum in 6-well plates coated with the PX004 extra-cellular matrix (AbCys, Paris, France). This matrix is produced by a human malignant epithelial cell line [12]. Contamination by murine fibroblasts was reduced by short applications of trypsin-EDTA taking advantage of stronger adherence of malignant epithelial cells to the culture support.

### Microsatellite analysis

For fingerprint experiments, DNA has been isolated from cell pellets using the DNeasy tissue kit (QIAGEN, Hilden, Germany), according to the manufacturer's instructions. Microsatellite genotypes have been established using twelve highly polymorphic dinucleotide microsatellite markers covering six different chromosome arms (5q, 8p, 17p, 17q, 18p, 18q). The sequences of the primers are from the Ensembl Genome System website [13]. PCR were carried out in 20 μL using 0.2 mmol/L dNTP, 0.5U HotStarTaq polymerase (QIAGEN), and sense primers labelled with 6-FAM (D5S107, D8S1731, D17S796, D17S1824, D18S53), HEX (D17S1353, D17S1791, D17S1873, D17S250, D18S1132) or NED (D8S261, D18S1127). After a 15-min step at 95°C, 35 cycles of 30 sec at 94°C, 30 sec at 55°C and 30 sec at 72°C, followed by a 6-min final extension at 72°C, were performed. PCR products were diluted in formamide containing ROX-labelled 400 HD size markers (PE Applied Biosystems) and electrophoresed in 50-cm capillaries containing POP-6 on an ABI PRISM^® ^3100 Genetic Analyzer (PE Applied Biosystems). The apparent sizes of the alleles were analyzed using the GeneScan Analysis 3.1 software (PE Applied Biosystems).

### Immunohistology

Tissue fragments were paraffin-embedded after fixation in 4% formol-PBS. Tissue sections were stained with antibodies directed to EMA (Epithelial Membranous Antigen, E29 MoAb, Dako), CEA (carcino-embryonic antigen, polyclonal, Dako), CK7 (OV-TL12.30 MoAb, Dako), CK20 (K020.8 MoAb, Dako), TTF1 (8G763/1 MoAb, Dako), EGF-R (clone 3C6, Ventana) and c-kit (D117 MoAb, Dako).

### Electron microscopy imaging

Cell pellets were fixed with 1.6 % glutaraldehyde at 4°C, followed by treatment with osmium tetroxide, then dehydrated and embedded in epon resin. Ultrathin sections were cut on an LKB-III ultra-microtome, stained for contrast with uranyl acetate and lead citrate and examined with a Zeiss EM 902 transmission electron microscope.

### Cytogenetics

Metaphase chromosome spreads were obtained using classical procedures, and standard karyotypic analyzes were performed after RHG banding and were classified according to ISCN classification [14]. FISH analysis was performed using 24 color multifish Vysis probes according to manufacturer's instructions (Vysis Downers Grove, Illinois, USA).

### Detection of *TP53 *gene mutations

Total DNA was extracted from xenografted tumor pieces. Exons 5 to 9 were amplified by PCR in three segments and analyzed by denaturing high performance liquid chromatography (DHPLC) followed by direct sequencing of regions generating abnormal chromatograms [15]. Corresponding PCR products were reamplified using nested primers and sequenced.

## Results

### Characteristics of donor patients

Transplantation into immunodeficient mice was attempted with fragments of tumor biopsy or surgical pieces from 4 patients. Their main clinical and histopathological characteristics are summarized in Table 1. For all these patients, a primary tumor had remained undetectable after the initial standard work-up including chest radiography, abdomino-pelvian ultrasound and CT scan, mammography for female patients, serum assays of PSA and β-HCG for male patients. Patient 1 (F, 34) presented with mediastinal and retroperitoneal lymph node metastases of a poorly differentiated adenocarcinoma with high plasma levels of CEA, CA125, CA 19-9 and no elevation of β-HCG or AFP. No lesions were detected by gynecological explorations. Despite a partial and transient response to chemotherapy, the issue was fatal in 3 months. Patient 2 (F, 66) presented with isolated pleural effusion revealing pleural metastases of a mucinous adenocarcinoma without lesions of lung parenchyma or any other primary tumor. She was treated by pleural symphysis and chemotherapy. Relapse in the pleura and homolateral lung occurred 2 years later with continued progression despite several lines of chemotherapy. Patient 3 (M, 46) was referred to our hospital for cervical lymph node metastases of a squamous cell carcinoma without a detectable primary tumor. He was treated by cervical dissection and radiotherapy but relapsed two years later in the mammary gland and axillary lymph nodes. Mastectomy and node dissection combined to chemotherapy could not prevent mediastinal extension and fatal issue. Patient 4 (M, 61) was treated for an isolated femur metastasis of a mucinous adenocarcinoma. After chirurgical resection, osteosynthesis and local radiotherapy, he remained in complete remission for 10 months. Then additional malignant lesions became apparent in the lung and adrenal gland. This patient is still alive after 3 years of evolution with slow progression under a third line of chemotherapy (vinorelbine, carboplatin).

**Table 1:** Characteristics of donor patients and outcome of mouse transplantation

	**Gender/Age**	**Site of tumor biopsy**	**Histological diagnosis**	**Outcome of xenotransplantation**
**Patient 1**	F 34	skin	poorly differentiated adenocarcinoma	successful
**Patient 2**	F 66	pleura	mucinous adenocarcinoma	unsuccessful
**Patient 3**	M 46	mammary gland	squamous cell carcinoma	unsuccessful
**Patient 4**	M 61	tibial bone	mucinous adenocarcinoma	successful

### Outcome of xenotransplantations and establishment of the Capi3 cell line

As shown in Table 1, mouse transplantation was successful in 2 cases out of four, resulting in the growth of the Capi1 (patient 1) and Capi3 (patient 4) xenografts. Capi1 was lost after 2 passages. However, it was possible to save frozen pieces of xenografted tumors. Capi3 xenografted tumor pieces were collected at passage 2 and tumor cells were released *in vitro *by mechanical dissociation. Dispersed tumor cells were then seeded for *in vitro *cultures as reported in the Methods section. Cell attachment was readily obtained on PX004 extra-cellular matrix, allowing slow growth of human epithelial cells mixed with murine fibroblasts. Beyond passage 5, it was possible to grow these cells on non-coated ordinary plastic vessels. Subsequently a progressive increase in cell growth was observed. At passage 20, doubling time stabilized at 4 days.

### Microsatellite genotyping at the successive stages of Capi3 establishment

**Figure 1:**
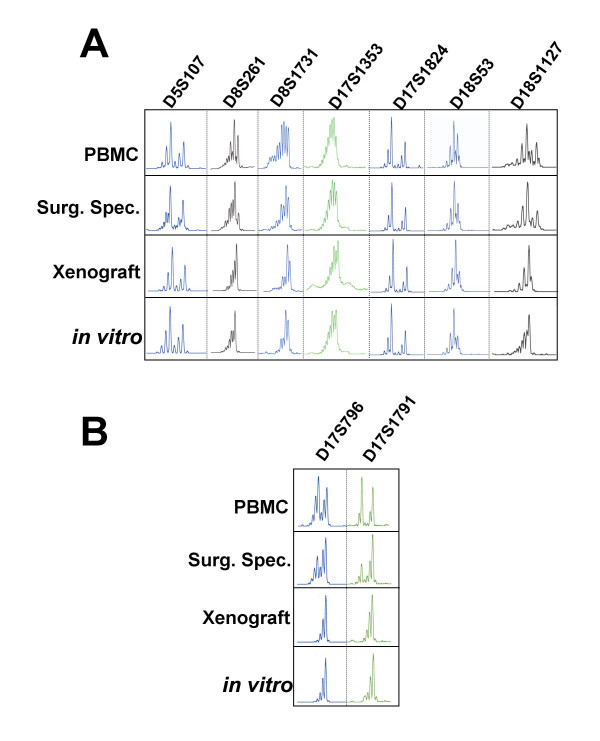
Microsatellite tracking assay linking DNA from patient peripheral blood mononuclear cells (PBMC), tumor surgical specimen (Surg. Spec.) and xenograft to the *in vitro *Capi3 cell line.

Microsatellite analysis was performed on various samples related to the establishment of Capi3 : DNA from donor patient peripheral blood mononuclear cells, initial tumor biopsy, xenografted tumor pieces and cells grown *in vitro*. All samples shared identical alleles at the 12 tested loci indicating that they were derived from a single individual. The results obtained with 7 markers are shown in Figure 1A. In addition to the bands corresponding to the allele sizes, the migration profiles of the CAn microsatellite markers contain extra bands that reflect slippage of Taq polymerase during PCR (-2 bp bands) and non-templated addition of an A residue (+1 bp bands). These results exclude the possibility that cross-contamination occurred at establishment of the xenograft or during *in vitro *cell propagation. In addition, the analysis of polymorphic markers located at 17p12 (D17S1791) and 17p13.1 (D16S796) where the *TP53 *gene is located demonstrates loss of heterozygosity (LOH) (Figure 1B). The fact that LOH is partial in the surgical specimen is likely due to the presence of normal cells contaminating the sample; alternatively, it may also reflect tumor heterogeneity. Both the xenograft and the cell line exhibit complete LOH at the *TP53 *region, in keeping with the presence of a *TP53 *mutation in this tumor (see subsequent paragraph).

### Histology, immuno-phenotyping and ultrastructure

**Figure 2:**
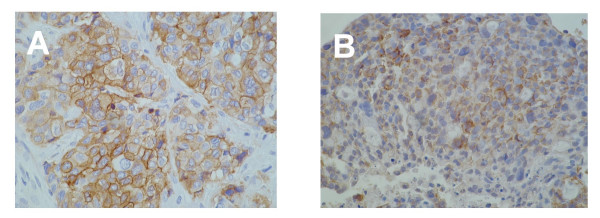
Detection of the c-kit tyrosine-kinase in Capi3 tissue sections. Panel A : surgical specimen (× 200). Panel B: xenografted tumor (× 100). Note that specific staining is mainly associated with the plasma membrane of malignant cells.

**Figure 3:**
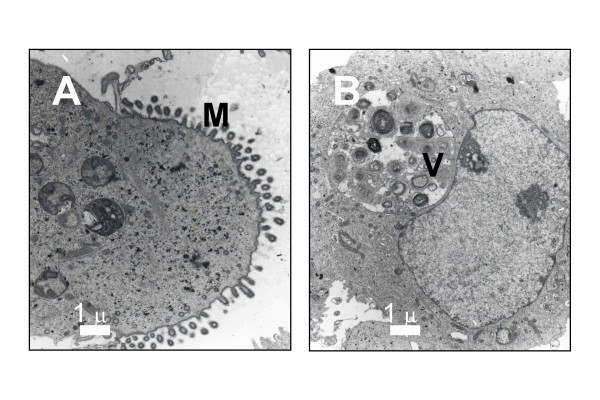
Electron microscopy of Capi3 cells propagated *in vitro*. **M**: microvillosities; **V**: large vacuole suggestive of aberrant autophagy.

The Capi1 xenograft was derived from a poorly differentiated carcinoma with a fibrous stroma. The same cellular morphology was found in the xenograft tissue sections although without the same stroma. A diagnosis of adenocarcinoma was suspected on the basis of morphological examination and confirmed by Alcian blue staining which demonstrated intra-cytoplasmic mucin (not shown). The Capi3 xenograft was derived from a mucinous adenocarcinoma with obvious aspects of secretory differentiation. The same cellular morphology was found in xenograft tissue sections. The expression of 6 protein tumor markers was investigated by immunohistology on the Capi1 and 3 xenografts and corresponding clinical specimens (see Table 2). Both xenografted tumor lines had abundant expression of EMA, CEA and cytokeratin 7 (CK7). CK 20 and TTF1 were negative on all tissue sections related to Capi1. In contrast, partial expression of these markers was found in Capi3 material. CK20 was weakly positive in some cells of the surgical piece whereas it was completely negative in the xenograft. Conversely, TTF1 was completely negative in the surgical specimen and weakly positive in the xenograft. Expression of the tyrosine-kinase receptors EGF-R and c-kit were investigated in both tumor lines. EGF-R expression was detected in the Capi1 biopsy but not in the corresponding xenograft. It was completely absent in Capi3 material. In contrast, the c-kit receptor was undetectable in Capi1 tissue sections but abundant in Capi3 material with a plasma membrane distribution suggesting that it was biologically active (Figure 2). Capi3 cells propagated *in vitro *were further characterized at the ultrastructural level (Figure 3). Glandular differentiation was confirmed by the polarized structure of many cells with abundant microvillosities at one pole (Figure 3A) and the observation of numerous clusters of glycogen granulations. The presence of large vacuoles (Figure 3B) containing multi-membranous structures suggested aberrant processes of autophagy.

**Table 2:** Immunohistological phenotyping of xenografted CUPs and corresponding clinical specimens

	**Capi1 clinical specimen**	**Capi1 xenograft**	**Capi3 clinical specimen**	**Capi3 xenograft**
**EMA**	90% ++	100% +++	100% +++	90% +++
**CEA**	100% +++	100% +++	100% +++	100% +++
**CK7**	100% +++	100% ++	100% ++	100% ++
**CK20**	negative	negative	30% +	negative
**TTF1**	negative	negative	negative	10% ++
**EGF-R**	90% ++	negative	negative	negative
**c-kit**	negative	negative	70% +	50% +++

Values correspond to the percentage of positive cells and the staining intensity.

### Cytogenetics

Very complex karyotypes were obtained in both tumor lines.

Capi1 was 59–60, XX, add(2)(q14), der(4)t(4;21)(pter->q28-q31::q24->q28-q32;q11), add(5)(p15), +6, +7 × 2, +8 × 2, -9 × 2, +11, +15, der(16)dup(16)(p12p13?, q22q23?), +der(17), +der(19), +der(20)(p11qter), -21, +mar cps.

**Figure 4:**
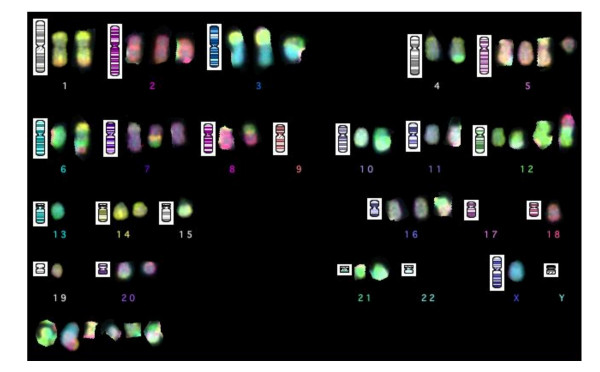
24 colour multifish karyotype showing the complexity of the genomic rearrangements with rearranged chromosomes in most pairs and several unidentified marker chromosomes (bottom left). Of particular interest are the translocation of chromosome 21 (in green) to the distal chromosome 4 (in grey) and the loss of chromosomes 9. In this cell, there were 2 der(3)t(3, 15) instead of one in most other cells which were analyzed.

Capi3 was 49–50, X, -Y, der(1)t(1;9) × 2, +2 × 2, der(3)t(3;15)(p23;q11), der(3)t(1,3;6), t(4;21)(q32-35; q11), +5, +der(5)t(5;21)(q11;q21), der(6)t(6;14), ider(6)t(6;18), der(7)t(7;16)(p22;q?), der(7)t(6;7;9)(p22;q?), der(8)t(8)t(8;10)(p11;p11), -9 × 2, der(10)t(10;13)(q10;q10), der(11)t(3;11)(q21;q23->q13::q23->pter), i(12q), -13, +der(15)t(3;15)(p23;q21), der(16)(t(16;22?)(q11;q11), der(17)t(17;19)(q11;q12); del(18)(q11q23), der(20)t(10;20)(p11;q13), der(22)t(8;12;22), + mar cps (see Figure 4).

It is noteworthy that a t(4;21) and a loss of both chromosomes 9 were observed in the two cell lines.

### *TP53 *gene status in Capi1 and Capi3

In Capi1, a C to G transversion was found at nucleotide 14696 of the *TP53 *locus (accession number X54156 in Genbank), in exon 9, resulting in a change of a threonine into a serine at codon 312. In Capi3, a G to C transversion was found at nucleotide 13221, in exon 5, resulting in a change of an arginine into a proline at codon 181.

## Discussion

In this study xenografting was successful for 2 out of 4 CUP specimens. This suggests that there is no real biological hindrance opposing successful grafting of CUP cells on immunodeficient mice. If CUP xenografts and cell lines remain so rare, it is probably due to a lack of deliberate efforts to obtain, preserve and spread this type of biological resource.

Regarding CK20 and TTF1 expression in Capi3, some differences were recorded between the surgical tumor specimen and the corresponding xenograft. Thanks to microsatellite analysis, we can rule out errors in tumor tracking and state positively that these differences reflect a genuine biological process, for example a selection of malignant cells more prone to grow in the murine microenvironment or a direct influence of this microenvironment on gene expression profile as reported in other models [16,17]. The same factors probably underlie the changes in EGF-R expression which is absent in the Capi1 xenograft while it was abundant in the corresponding clinical specimen.

The karyotypes of both cell lines exhibited very complex chromosomal rearrangements. This is consistent with a previous report by Pantou et al. which has shown that most CUPs – although not all of them – are associated with massive structural alterations of chromosomes [18]. Additional investigations such as high resolution array CGH will be required to better understand those complex rearrangements. One important aim will be to elucidate the t(4;21) translocation observed in both Capi1 and Capi3. So far t(4, 21) translocations have been exclusively reported in hematological malignancies according to the "Mitelman database of chromosome aberrations in cancer", especially in myeloid leukemias [19].

There has been only one previous study on *TP 53 *mutations in CUPs which analyzed exons 5 to 9 in 23 CUP specimens and found only 6 tumors with mutations (26%); a frequency which was lower than expected for this aggressive type of epithelial malignancy. One of the 6 positive specimens had a mutation at nucleotide 13 221 (Bar-Eli *et al.*, 1993)[20]. In our own study, the same nucleotide was mutated in the Capi3 tumor. Both mutations resulted in a missense alteration at codon 181 : Arg to Leu (Bar-Eli et al.) and Arg to Pro (Capi3) respectively. Mutations of codon 181 are relatively rare, accounting for only 59 cases out of 23 544 somatic mutations indexed in the IARC database (to be compared to 1130 mutations recorded for codon 175; for details see the "IARC *TP 53 *mutation data base", release R11, 2006) [21-23]. P53 amino-acid 181 is contained in the DNA-binding domain but has no direct contact with the DNA in contrast with residues identified as mutational hot spots. Nevertheless, functional studies have shown that both Leu181Arg and Pro181Arg mutations impair the transactivating functions of TP 53 [24,25]. It would be interesting to investigate additional CUP specimens to know whether mutations of codon 181 occur at a relatively high frequency in this category of malignancies. A distinct *TP 53 *mutation was found at codon 312 (Thr to Ser) in the Capi1 xenograft. This codon accounts for only 13 cases of somatic mutations recorded in the IARC data base. The affected amino-acid is outside known functional domains. According to prediction models, this mutation is not expected to be deleterious (IARC *TP 53 *mutation data base, R11, 2006) [21-23]. No mutation of exon 9 have been reported in the previous study on *TP 53 *mutations in CUPs [20].

Intense membrane expression of c-kit was found in Capi3 cells in the xenografted tumor as well as in the surgical specimen. We and others have found that the c-kit protein is detected in about 10% of CUP specimens [2,26]. Using the c-kit tyrosine kinase inhibitor, imatinib mesylate, remarkable therapeutic results have been achieved in human malignancies overexpressing a mutated form of c-kit, especially GISTs. In contrast, this compound has only limited effects on malignancies expressing a non-mutated form of c-kit, for example small cell lung carcinomas [27]. However a novel generation of c-kit inhibitors which are currently under investigation might be efficient on a larger spectrum of c-kit positive malignancies [28]. From this perspective, the Capi3 cell line will probably be a useful target for *in vitro *assessment of these novel compounds.

## Conclusion

This report will encourage other investigators to attempt establishment of CUP cell lines in order to broaden our possibilities of laboratory investigations. *TP 53 *mutations might be more frequent than initially suspected in this disease [20]. The hypothesis of a preferential association of CUPs with mutations of codon 181 deserves further investigations. Finally, the Capi3 cell line will be a useful tool for assessment of novel c-kit inhibitors.

## Competing interests

The author(s) declare that they have no competing interests.

## Authors' contributions

DL carried out xenografts and cell cultures and was involved in most other studies. KF provided clinical specimens and participated in the design of the study. ST made karyotype analyzes. SS made electron microscopy experiments and observations. MCM supervised histological examination and immuno-phenotyping. PH made *TP 53 *gene analysis. AB contributed to karyotype analysis. FP made microsatellite genotyping and contributed to draft the manuscript. PB conceived the study and its design and wrote the manuscript. All authors read and approved the final manuscript.

## Pre-publication history

The pre-publication history for this paper can be accessed here:


